# Stereotactic Body Radiation Therapy for the Management of Hepatocellular Carcinoma: Efficacy and Safety

**DOI:** 10.3390/cancers14163892

**Published:** 2022-08-11

**Authors:** Isaure Roquette, Emilie Bogart, Thomas Lacornerie, Massih Ningarhari, Jean-Emmanuel Bibault, Marie-Cecile Le Deley, Eric F. Lartigau, David Pasquier, Xavier Mirabel

**Affiliations:** 1 Academic Department of Radiation Oncology, Centre Oscar Lambret, 59020 Lille, France; 2 Department of Biostatistics, Centre Oscar Lambret, 59020 Lille, France; 3 Department of Medical Physics, Centre Oscar Lambret, 59020 Lille, France; 4 Department of Hepatogastroenterology, CHU Lille, 59020 Lille, France; 5 Lille Inflammation Research International Center–U995, Lille University, Inserm, CHU Lille, 59020 Lille, France; 6 Inserm UMR 1138 Lab, Centre de Recherche des Cordeliers, Medical Informatics Unit, George Pompidou European Hospital, 75006 Paris, France; 7 Radiation Oncology Department, Georges Pompidou European Hospital, 75015 Paris, France; 8 CRIStAL UMR CNRS 9189, Lille University, 59000 Lille, France

**Keywords:** stereotactic body radiation therapy, hepatocellular carcinoma, SBRT, HCC, radiotherapy

## Abstract

**Simple Summary:**

This study aimed to describe treatment efficacy and safety in patients with hepatocellular carcinoma (HCC) undergoing stereotactic body radiation therapy (SBRT). In one of the largest retrospective studies to date, we analyzed the data of 318 patients. The median follow-up period was 70.2 months. The local control rate at 24 and 60 months was 94% (91–97%) and 94% (91–97%), respectively. Relapse-free survival at 12, 24, and 60 months was 62% (55–67%), 29% (23–36%), and 13% (8–19%), respectively. OS at 12, 24, and 60 months was 72% (95%CI 67–77%), 44% (38–50%), and 11% (7–15%), respectively. The outcome is highly related to the natural evolution of the underlying cirrhosis. Child-Pugh score B-C, high BCLC score, portal thrombosis, GTV volume, and higher PTV volume reported on total hepatic volume ratio were significantly associated with OS. SBRT is efficient for the management of HCC with a favorable toxicity profile.

**Abstract:**

This study aimed to describe patient characteristics, treatment efficacy, and safety in patients with hepatocellular carcinoma (HCC) undergoing stereotactic body radiation therapy (SBRT). We retrospectively analyzed data of 318 patients with 375 HCC treated between June 2007 and December 2018. Efficacy (overall survival [OS], relapse-free survival, and local control) and acute and late toxicities were described. The median follow-up period was 70.2 months. Most patients were treated with 45 Gy in three fractions. The median (range) PTV volume was 90.7 (2.6–1067.6) cc. The local control rate at 24 and 60 months was 94% (91–97%) and 94% (91–97%), respectively. Relapse-free survival at 12, 24, and 60 months was 62% (55–67%), 29% (23–36%), and 13% (8–19%), respectively. OS at 12, 24, and 60 months was 72% (95%CI 67–77%), 44% (38–50%), and 11% (7–15%), respectively. Approximately 51% and 38% experienced acute and late toxicity, respectively. Child-Pugh score B-C, high BCLC score, portal thrombosis, high GTV volume, and higher PTV volume reported on total hepatic volume ratio were significantly associated with OS. SBRT is efficient for the management of HCC with a favorable toxicity profile. The outcome is highly related to the natural evolution of the underlying cirrhosis.

## 1. Introduction

The incidence of hepatocellular carcinoma (HCC) has been increasing over the past few years. It is now the third most common cause of cancer-related deaths worldwide [1]. HCC usually occurs in the context of cirrhosis, which is mainly caused by a viral infection or chronic alcoholism. However, the emergence of non-alcoholic fatty liver disease (NAFLD) due to lifestyle changes leads us to believe that the number of cases will continue to rise.

With surgery (resection or liver transplantation) or percutaneous destruction (ethanol injection or radiofrequency), HCC should be curable [2,3]. However, due to comorbidities or characteristics of the disease itself (i.e., its anatomical relations), many patients cannot benefit from these treatments. In these cases, other treatments have proven beneficial. Chemoembolization has improved survival rates [4], and is recommended as the first-line treatment for advanced HCC in patients with preserved hepatic function, good Eastern Cooperative Oncology Group (ECOG) performance score, no metastases, and no abnormality of portal flow [5,6]. Since 2007, targeted therapies have emerged as possible palliative treatments for HCC. The kinase inhibitors sorafenib and lenvatinib have been shown to improve survival versus placebo [7,8], and appear to be a good treatment option for patients ineligible for specific treatments or after failure of the treatment [9]. More recently, the results of the Check Mate 040 and Keynote 224 studies have permitted nivolumab and pembrolizumab, respectively, to obtain FDA approval for the treatment of advanced HCC [10,11]. After the publication of the results of the IMbrave150 trial demonstrating an overall survival benefit compared to sorafenib, the combination of atezolizumab with bevacizumab has become a standard in France in advanced HCC [12].

Stereotactic body radiation therapy (SBRT) has also appeared to be a possible ablative treatment for HCC. To date, this therapy has been evaluated in retrospective and phase 2 studies [13,14,15,16,17]. These have shown promising results, with good local control, survival rates, and a favorable toxicity profile. Despite these advantages, SBRT remains mainly proposed for HCC that is inaccessible to curative treatment and after the failure of other therapies.

SBRT is a technique that has been developed in our center in 2007, with a large regional recruitment since then. This gave us the opportunity to gather a large cohort of patients homogeneously treated, with a unique epidemiology regarding the cause of cirrhosis, allowing us to provide evaluation data on SBRT in this disease.

The aim of this retrospective study was to describe the population characteristics and to estimate the safety and efficacy in terms of overall survival (OS), relapse-free survival (RFS), and local control in a large cohort of patients treated with SBRT for HCC, since the establishment of the technique in our center. We also aimed to identify prognostic factors associated with OS and toxicity after SBRT. Finally, we examined the pathological response of patients treated with SBRT as a bridge to liver transplantation.

## 2. Methods and Materials

### 2.1. Patients

All patients who started SBRT on hepatic lesions in our center between June 2007 and December 2018 were retrospectively included in the current study, leading to a total of 318 patients. The patient selection was performed using the CONSORE tool.

The diagnosis of HCC was made using liver biopsy or dynamic imaging, according to Barcelona diagnosis criteria [18]. In this case, HCC diagnosis was established by the detection of contrast enhancement in the arterial phase (wash-in), followed by hypoenhancement in the portal phase (wash-out) on magnetic resonance imaging and/or computed tomography. Indication of SBRT was based on the decision by the institutional multidisciplinary consultation committee of hepatic tumors, which considered among other factors, patient comorbidities, ECOG score, anatomical relations of the HCC, and multiplicity and site of lesions. SBRT was discussed for patients with up to four synchronous hepatic lesions without distant lesions. Failure of other local treatments, such as radiofrequency (RFA) or trans-arterial chemoembolization (TACE) was also an indication for SBRT. Patients could have previously undergone surgical resection, RFA, TACE, radiotherapy, chemotherapy, and targeted therapy. Any previous treatment was not recorded if the target lesion was different from the one treated with SBRT. Child-Pugh, Model For End-Stage Liver Disease (MELD) and Barcelona Clinic Liver Cancer (BCLC) scores were calculated based on clinical, biological and imagery data collected during the first consultation with the radiotherapist. All clinical data were collected from computerized patient records.

The study complies with the “reference methodology” adopted by the French Data Protection Authority (CNIL), all patients provided informed consent for data collection and analysis during the follow-up survey and the study was approved by the relevant Institutional Ethics Committee.

### 2.2. Treatment

For each patient, hepatic SBRT therapy with Cyberknife^®^ was preceded by placement of fiduciaries to track the lesions. Our process has been previously described in Jarraya et al. [19]. Planning CT is realized during a brief apnea. Additionally, if possible, patients underwent MRI, which was registered with CT, when fiducials were visible on MR, to help with delineation of the target volumes and organs at risk, performed by a radiation oncologist. T2 and dynamic portal venous phases were used to delineate tumors. Clinical Target Volume (CTV) was defined as Gross Target Volume (GTV) + 5 mm margins and Planning Target Volume (PTV) as CTV + 3 mm margins. For multiple lesions, the nearby targets were merged into the same volume.

A total dose of 21–54 Gy in 3–6 fractions was prescribed on the 80% isodose line. Dose to organs at risk (duodenum, stomach, and liver) adhered to the dose constraints in effect at our center. If necessary, target volumes coverage, the total dose, and the number of fractions was modified to fulfill these constraints (from TG 101 AAPM [20]) and adapted for six fractions. In rare cases, the CTV could be suppressed to meet the dose constraints. Dosimetry data was collected from Accuray Precision^®^ software according to International Committee for Radiological Units (ICRU) 91 criteria [21].

Intra-fraction motion management was dealt with the Synchrony^TM^ system, which correlates the movement of the fiduciaries with external diodes placed on the patient’s chest. This enables the system to follow translations of the target during free breathing The use of an abdominal compression belt could be combined to limit respiration-induced movements to facilitate the creation of a correlation model.

### 2.3. Follow-Up

Follow-up was planned every three months in the first three years, and every six months afterwards. A clinical examination was performed by either a radiation oncologist or gastroenterologist. Each consultation was preceded by imaging (MRI or CT scan). Responses were assessed according to the modified RECIST (mRECIST) criteria for hepatocellular carcinoma [22]. Blood work was also performed, collecting data on toxicity and alpha fetoprotein levels. Post-treatment Child-Pugh scores and alpha fetoprotein levels were recorded at three months post-treatment, which corresponded to the first medical follow-up consultation.

We also reported whether the patient had undergone liver transplantation after SBRT; for these patients, the pathological response was evaluated by analyzing the explanted liver. Complete response was defined as complete necrosis of the lesion without any remaining malignant cells.

### 2.4. Endpoint Definition

OS was estimated from the start of treatment until death from any cause. Patients alive at the date of the last news event were censored at this date. RFS was estimated from the start of treatment until the date of the first relapse (local, hepatic or distant) or death from any cause. Patients alive at the date without relapse of the last news were censored at this date. Local control was estimated from the start of the treatment until the first local relapse. Other types of relapse and death were considered competitive events.

Toxicities were graded using the Common Terminology Criteria for Adverse Events v5.0. Acute toxicity was defined as adverse events occurring in the first three months following treatment, based on the first follow-up visit; while late toxicity was defined as events occurring after at least three months of follow-up.

### 2.5. Statistical Analysis

The median follow-up was estimated using the reverse Kaplan–Meier method. OS and PFS curves were estimated using the Kaplan–Meier method. To describe the local control rate, we estimated the cumulative incidence of local recurrence using Kalbfleisch and Prentice method. The association between the risk of death and prognostic factors was analyzed using a Cox model after checking the proportional hazard assumption. The association between prognostic factors and acute toxicity of grade ≥2 and late toxicity of grade ≥2 was analyzed using logistic regression models. The software used for the statistical analysis was Stata version 15.0 (StataCorp. 2017. Stata statistical software: Release 15 StataCorp LLC, College Station, TX, USA).

## 3. Results

### 3.1. Patient and Treatment Characteristics

A total of 318 patients were treated, for 375 lesions. Fourteen patients received a further SBRT for a new lesion. The patient characteristics are described in Table 1. The median (range) age was 69 (43–93) years. Approximately 85% (*n* = 269) of the patients were men. The most common cause of cirrhosis was chronic alcoholism (59%, *n* = 160); other causes included multifactorial causes (13.6%, *n* = 37), viral (12.1%, *n* = 33), and NAFLD (10.3%, *n* = 28). Most patients had Child A cirrhosis (86%, *n* = 204), and one patient had Child-Pugh C cirrhosis. The median (range) MELD score was 9 (6–19). The BCLC stage was A in nearly half of the patients (47%, *n* = 128). The median (range) alpha fetoprotein level was 9.8 (0.9–44,286) ng/mL. Approximately 16.97% (*n* = 40) had portal vein thrombosis. Seventy-five percent (*n*= 249), and 20% (*n* = 66) of patients had single HCC, and two synchronous lesions, respectively. Seventeen (5%) patients had three lesions and two patients (0.6%) had four lesions. The median (range) tumor size was 30 mm (5–105) mm.

Approximately 32% (*n* = 100) of patients had received previous local treatment for targeted HCC. This treatment included chemoembolization in 68 patients (22%), radiofrequency in 28 patients (9%), surgical resection in 16 patients (5%), and radiotherapy in two patients (0.6%). Twenty-three patients (7%) had previously received targeted therapy; 29 had received at least two previous treatments, of whom 16 had received at least two previous local treatments, most frequently chemoembolization and radiofrequency.

The median (range) total dose prescribed was 45 (15–54) Gy, with a median (range) dose per fraction of 15 (3.5–15) Gy. Most patients received three fractions (299 patients, 95%), one patient (0.3%) received four fractions, two patients (0.6%) received five fractions, and 14 patients (4.4%) received six fractions. Treatment was interrupted prematurely in two patients (0.6%). Considering all 375 treated lesions individually, the median (range) GTV volume, CTV volume, and PTV volume was 21.1 (0.4–700.1) cc, 63.3 (2.5–999.4) cc, and 90.7 (2.6–1067.6) cc, respectively. D98%, D50%, and D2% of the PTV were 43.6 (13.3–52.4) Gy, 48.9 (23.4–63.2) Gy, and 52.3 (25.1–74.8) Gy, respectively. The ratio of PTV volume reported on total hepatic volume was less than 10% for 66% of patients, with a median of 7%. The median (range) maximum dose received by 700 cc of the liver was 9.2 (0.4–34.1) Gy. The dosimetry characteristics are described in Table 2.

### 3.2. Efficacy

**Figure 1:**
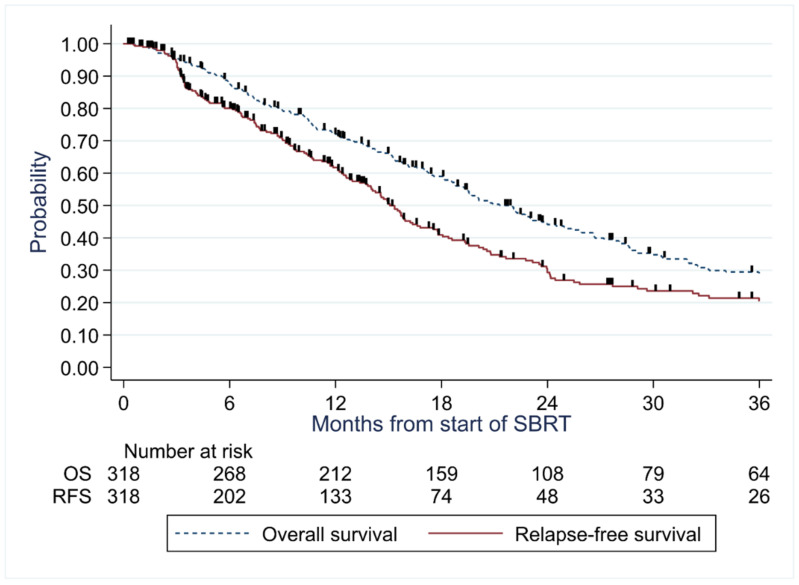
Overall survival and relapse-free survival for the general population.

**Figure 2:**
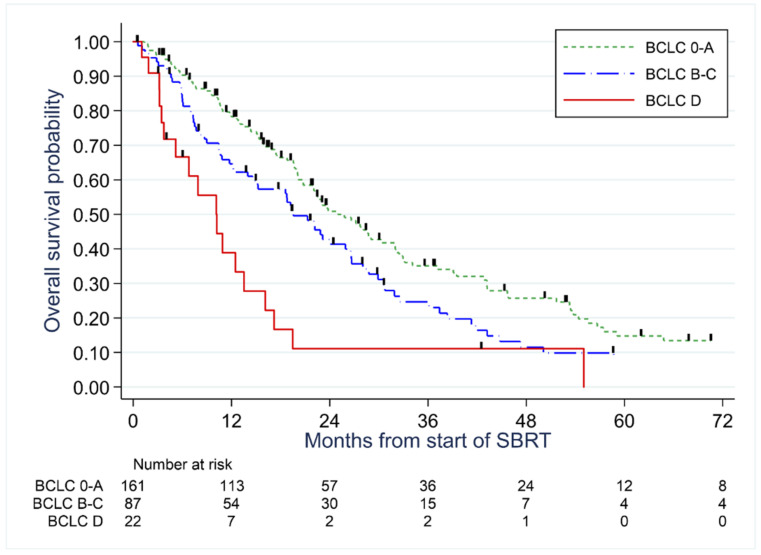
Overall survival probability according to the BCLC stage.

The median follow-up duration was 70.2 months. At the time of the analysis, 240 deaths were observed, with a median OS of 22 months (95%CI, 19–24 months), and 12-month, 24-month and 60-month rates were 72% (67–77%), 44% (38–50%), and 11% (7–15%) (Figure 1). We observed a decreasing probability of overall survival with an increasing Barcelona score, with a hazard ratio of death of 1.40 (95%CI, 1.04–1.90) and 2.94 (1.78–4.88) in patients with scores B-C and D respectively compared to patients 0-A (*p* < 0.001) (Figure 2).

**Figure 3:**
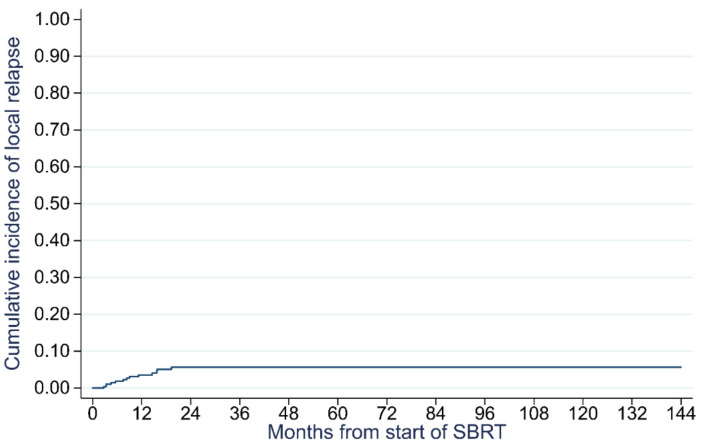
Cumulative incidence of local relapse.

Thirteen local relapses were observed, leading to 12-month, 24-month, and 60-month local control rates of 97% (94–98%), 94% (91–97%), and 94% (91–97%), respectively. Figure 3 illustrates the cumulative incidence of local relapse.

The median RFS was 15 (14–17) months and 12-month, 24-month and 60-month rates were 62% (55–67%), 29% (23–36%), and 13% (8–19%), respectively (Figure 1).

Considering all evaluations performed during follow-up, there was complete response in 156 patients (60%), partial response in 70 patients (27%), and stable disease in 24 patients (9%). Eleven patients did not respond to treatment (4%).

### 3.3. Safety

**Figure 4:**
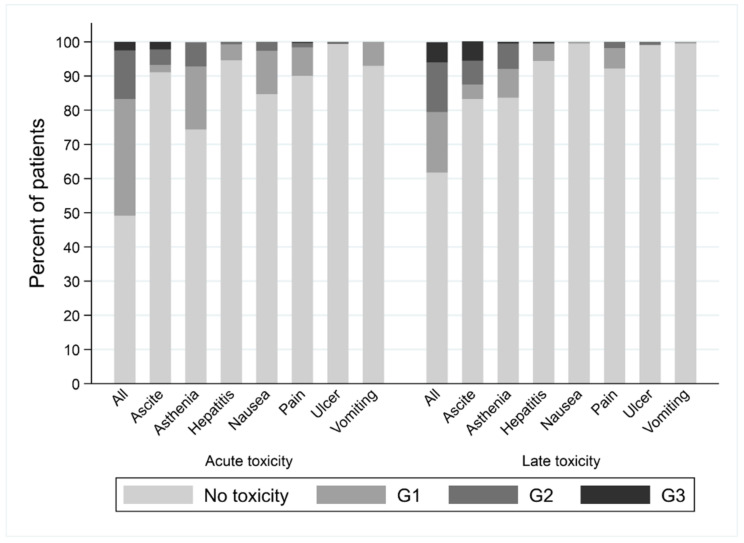
Description of acute and late toxicities according to the grade.

A total of 161 patients (51%) experienced acute toxicity; 108 (grade 1), 45 (grade 2), and 8 (grade 3). The most frequent toxicities were fatigue (79 patients, 26%, including grade 2 or higher in 22 patients) and nausea (47 patients, 15%, including grade 2 or higher in 8 patients). No grade > 3 acute toxicities were observed (Figure 4).

Eighty-four patients (38%, 98 missing data) experienced late toxicity; 39 (grade 1), 32 (grade 2), and 13 (grade 3). The most frequent late toxicities were fatigue (35 patients, 16%, including 17 patients with grade 2 or higher) and ascites (36 patients, 17%, including 27 patients with grade 2 or higher). No grade > 3 late toxicities were observed.

Child Pugh score has been calculated after treatment in 97 patients. Child Pugh score was stable or improved in 53% of the patients. The score increased by 1, 2, and 3 points in 23%, 9%, and 9% of the patients, respectively. Toxicities are described in Table 3.

### 3.4. Analysis of Prognostic Value of Patient’s Characteristics and Outcomes According to Radiotherapy Parameters

As illustrated by Figure 2, univariate analyses of the overall survival showed that the risk of death was significantly associated with higher BCLC score (compared to BCLC 0: HR = 1.31 for BCLC A, 95%IC 0.80–2.13; HR = 1.52 for BCLC B, 95%IC 0.84–2.74; HR = 1.89 for BCLC C, 95%IC 1.11–3.21; HR = 3.64 for BCLC D, 95%IC 1.92–6.93, *p* < 0.001). We also observed a significant association with Child-Pugh score B or C (HR = 1.87, 95%CI 1.25–2.82, *p* = 0.003), presence of portal thrombosis (HR = 1.56, 95%IC 1.09–2.23, *p* = 0.02). However, both factors were no longer significantly associated with poor prognosis when adjusted for BCLC score (*p* = 0.11 and *p* = 0.67, respectively). On the other hand, we observed, both in univariate and multivariate analyses adjusted on BCLC, a significant association between poor prognosis and tumor volume when considering high GTV volume (HR/100 cm^3^ =1.22, 95%IC 1.09–1.35, *p* < 0.001 in univariate analysis; HR/100 cm^3^ =1.28, 95%IC 1.09–1.50, *p* = 0.002 when adjusted on BCLC), or high PTV volume reported on total hepatic volume ratio (HR/10% = 1.34, 95%IC 1.17–1.55, *p* < 0.001 in univariate analysis; HR/10% = 1.33, 95%IC 1.10–1.62, *p* = 0.004 when adjusted on BCLC). Other patient characteristics were not significantly associated with the risk of death (Table 4).

Grade ≥ 2 acute toxicity was significantly associated with higher pre-treatment bilirubin (OR_/10_ µmol/L = 1.31, 95%IC 1.04–1.65, *p* = 0.02), a high GTV volume (OR_/100_ cm^3^ = 1.34, 95%IC 1.04–1.73, *p* = 0.02) and high PTV volume reported on total hepatic volume ratio (OR_/10%_ = 1.63, 95%IC 1.17–2.3, *p* = 0.004). The other parameters were not significantly associated with acute grade ≥ 2 toxicity. (Table 4)

Grade ≥ 2 late toxicity was significantly associated with a low albumin level (OR_/10 g/L_ = 0.42, *p* = 0.026).

### 3.5. Response after Liver Transplant

Eleven patients (3%) benefited from liver transplantation. The median time between SBRT and transplantation was 13 months (range, 1.8–24.5). Based on the analysis of the explanted liver, six patients had a complete response to the targeted HCC and five had a partial response. The median time to transplantation for these five latter patients with a partial response was 5 months (range 1.9–13.1), whereas the median time to transplantation for patients with a complete response was 15.2 months (range 1.8–24.5). Pathological analysis revealed disease outside the target volume in eight patients.

## 4. Discussion

This study supports the fact that SBRT provides excellent local control for HCC, with an acceptable tolerance profile. In the current treatment guidelines, SBRT is not universally accepted as a first-line treatment for HCC, and is even absent in the recommendations of the European Association for the Study of the Liver (EASL) [2], the Japanese Society for Hepatology (JSH) [23] and the Asian Pacific Association for the Study of the Liver (APASL) [24]. However, the American Association for the Study of Liver Disease (AASLD) [3] and the European Society for Medical Oncology (ESMO) [25] guidelines mention SBRT as an option for early-stage HCC.

This study evaluated the efficacy and safety of SBRT for HCC in 318 patients with 375 lesions. To our knowledge, this is the largest retrospective study on this subject. Our cohort is unique as the prevalence of alcohol abuse in our population is considerably higher than in other studies, in which viral hepatitis has been identified as the main cause of cirrhosis. This factor increases the risk of tumor progression as well as deterioration of liver function, thus making our population more precarious. In addition, our retrospective study is the only one to extensively report dosimetry results according to the ICRU 91 recommendations. Our results support the fact that SBRT is a safe and efficient treatment option for unresectable HCC.

SBRT allows excellent local control, which has been demonstrated in previous retrospective studies. In their systematic review, Dobrzycka et al. [26] studied the outcomes of SBRT in early-stage hepatocellular carcinoma. Sixteen studies (973 patients with 1034 lesions), of which 14 were retrospective, were included. Treatment methods included Cyberknife (35%), intensity-modulated radiotherapy (IMRT) (35%), volumetric arc therapy (VMAT) (30%), and 3–6 fractions at a dose of 5–20 Gy were delivered. The mean weighted local control at 1, 2, and 3 years was 94%, 92%, and 93%, respectively. The mean OS rates were 90.9%, 67.5%, and 73.4% at 1, 2, and 3 years, respectively. However, as mentioned, this review included patients with early-stage HCC, with a mean tumor diameter of 23.15 mm and no tumor exceeding 5 cm. Other studies have shown the same tendency, with 2-year local control rates ranging from 77% to 97% [14,27,28,29]. Prospective studies on this subject are scarce [13,14,15,16,17,30,31]. In a recent phase-2 multicentric prospective study, Durand-Labrunie et al. evaluated the use of SBRT for newly diagnosed HCC [11,32]. Forty-three patients with single HCC deemed unsuitable for standard locoregional therapies received SBRT at a dose of 45 Gy in three fractions. The 18-month local control rate was 98% (95% CI 85–99%) and the 18-month OS rate was 72% (95% CI, 56–83%). Grade ≥ 3 acute adverse events were reported in 31% of patients, mainly abnormalities in liver function tests (21%), which was described as being higher than in other published series.

These outcomes in the present study were comparable to other local therapies. A meta-analysis found similar local control rates for SBRT and RFA [32], while others found better local control in the SBRT group [33,34]. TACE and SBRT have also been shown to be comparable in terms of LC and OS [35], although other studies found superior LC, PFS, and intrahepatic control in the SBRT group, with similar OS [36,37]. For medium-sized HCCs, SBRT appears to be superior to TACE [38]. For small HCCs with Child A cirrhosis, a matched-pair analysis found no difference in OS and PFS between SBRT and liver resection [39]. However, these findings should be validated in large phase III clinical trials.

In this study, we found that Child scores B or C, higher Barcelona Clinic Liver Cancer stage, the existence of portal thrombosis, PTV volume reported on total hepatic volume ratio, and larger GTV volume was significantly associated with a higher risk of death. The findings of previous studies confirm the reliability of these factors in predicting mortality [15,16,28]. These factors reflect more advanced HCC and underlying liver disease and highlight the importance of patient selection before SBRT. However, we must remember that these factors can also contra-indicate other therapies, or be the reason for their failure; in these cases, SBRT can be the only local treatment option. Other prognostic factors for OS found in the literature include radiation dose [40], albumin levels [17], and performance status [41]. Zhang et al. established a nomogram to predict mortality after SBRT for unresectable HCC, and identified race, T stage, N stage, M stage, and chemotherapy as independent risk factors for survival [42].

The uniqueness of our study lies in the very high proportion of patients with cirrhosis caused, at least partially, by alcohol abuse. However, this characteristic did not appear as a statistically significant prognostic factor of global survival or toxicity, whether or not alcohol consumption was pursued.

Given the small number of in-field recurrences in our study, we did not analyze the prognostic factors for local control. The main factors which have been found to be associated with local control were radiation dose and tumor size [28,41].

SBRT is a safe treatment option. In our cohort, no deaths were attributed to treatment toxicity, and we did not have any treatment interruption due to poor tolerance. Two of our patients did not complete the full treatment. One patient suffered from myocardial infarction after two fractions and died 12 days later, while the other patient presented with confusion and a suspicion of hepatic encephalopathy after one fraction and died three weeks later.

Most patients with acute toxicities present with mild symptoms. Seventeen percent of patients presented with grade ≥ 2 acute toxicities, none higher than grade 3, which is similar to what was found in previous studies [14,15,43]. As in the present study, the most commonly reported acute toxicities were fatigue, nausea, and hepatic pain.

We identified high GTV volume and high PTV volume reported on total hepatic volume ratio as prognostic factors of grade > 1 toxicity. We were not able to specifically study risk factors for each type of toxicity given the scarcity of events and the low reliability of retrospective data collection for some events such as fatigue.

Late toxicities were mainly fatigue and ascites. However, both can also be explained due to the natural progression of HCC and underlying cirrhosis, and identifying the contribution of SBRT in the occurrence of these symptoms remains a challenge. Moreover, the assessment of late toxicity is limited by the retrospective nature of the present study.

The main cause of failure after treatment was an out-field hepatic relapse, which has also been reported in previous studies on SBRT. This is explained by the fact that HCC is a multicentric disease that occurs from an underlying pathologic liver, creating a microenvironment favorable for carcinogenesis.

Future perspectives include the association of systemic treatment with local therapies. The emergence of immunotherapies for other indications has led to the question of their interest in the treatment of HCC. The FDA has approved the use of nivolumab and pembrolizumab for HCC previously treated with sorafenib, based on the result of the Check Mate 040 and KEYNOTE-224 studies [10,11]. In a recent phase III, randomized, double-blind trial, pembrolizumab was compared to placebo and tended to show an improvement in OS and PFS, but the results were not statistically significant [44]. The combination of checkpoint inhibitors with SBRT appears to be an interesting approach, with a rationale based on the antitumor immune responses of these therapies [45,46,47]. A case report of five patients treated with SBRT followed by anti-PD1 antibodies has shown promising results, with 1-year local control and OS of 100% [48]. Further investigations are needed, and trials are currently underway to explore this perspective [49].

Despite advances in local and systemic therapies, the best treatment for HCC remains liver transplantation. With graft shortage, the waiting time can be long, which is why local treatments can be indicated to temporarily prevent tumor progression. SBRT can also be proposed for downstaging to meet the Milan criteria [50]. In our cohort, 11 patients benefited from liver transplantation, and all had a partial or complete response of the target on the pathological analysis of the explanted liver. If we look at the five patients with partial response, their time to transplantation was inferior to the median of the whole cohort. It is possible that the surgical intervention was conducted before the lesion had reached complete necrosis. Out-field HCC was found in eight patients, which is consistent with the above-mentioned fact that HCC is a multicentric disease. Previous studies have found similar results with good local control rates and favorable toxicity profiles [14,51]. In a series of 38 patients treated with SBRT, the histologic response rate (complete plus partial response) was 68% [52]. In a prospective study, patients were enrolled for SBRT under a standardized protocol and compared with a retrospective cohort of patients who underwent TACE or High Intensity Focused Ultrasound (HIFU). One hundred fifty patients were evaluated (SBRT, *n* = 40; TACE, *n* = 59; HIFU, *n* = 51). The tumor control rate at 1 year was significantly higher after SBRT compared with TACE and HIFU (92.3%, 43.5%, and 33.3%, respectively; *p* = 0.02). With competing risk analysis, the cumulative incidence of dropout at 1 and 3 years was lower after SBRT compared with TACE and HIFU too. The authors concluded that SBRT was safe, reduced the risk of waitlist dropout, and should be used as an alternative to conventional bridging therapies [53]. This strategy has also shown its safety and efficacy in patients with more advanced Child-Pugh B or C cirrhosis [54,55].

The main limitations of this study are those inherent to retrospective studies, mainly incomplete data. Large prospective, phase III trials would be needed to confirm the evidence pointed out in our study and other previous ones.

## 5. Conclusions

SBRT is safe and efficient for the treatment of inoperable hepatocellular carcinoma or as a bridge to transplantation. Efficacy is comparable to that of other local therapies, thus, its indication must not only be evoked after the failure of these treatments, but should be considered at any stage in the management of HCC. The pattern of failure is proof of a multicentric disease, and further studies are needed to show the benefits of concomitant systemic therapies.

## Figures and Tables

**Table 1:** Description of the population at diagnosis (*n* = 318).

Demographic Data		
**Age**	69	(43; 93)
**Sex**		
Male	269	84.6%
Female	49	15.4%
**ECOG Performance status** (MD = 29)		
0	146	50.5%
1	106	36.7%
2	32	11.1%
3	5	1.7%
**BMI in kg/m^2^** (MD = 95)	28.9	(13.6; 48.1)
Underweight (<18.5)	3	1.3%
Normal build (18.5–25)	50	22.4%
Overweight (25–30)	77	34.5%
Obese (30–40)	78	35.0%
Morbidly obese (≥40)	15	6.5%
**Smoking** (MD = 142)		
Non-smoker	45	25.6%
Smoker	29	16.5%
Ex-smoker	102	58.0%
**Alcohol intoxication** (MD = 79)		
Non-drinker	33	13.8%
Drinker	58	24.3%
Former drinker	148	61.9%
**Diagnosis**		
**Type of diagnosis** (MD = 72)		
Histology	70	28.5%
Radiology	176	71.5%
**Cause of cirrhosis** (MD = 45)		
Alcohol	160	58.6%
Viral	33	12.1%
NASH	28	10.3%
Medication	1	0.4%
Hemochromatosis	11	4.0%
Combination	37	13.6%
Autoimmune	3	1.1%
**Cirrhosis-Alcohol** (MD = 20)		
No alcohol, cause of cirrhosis different from alcohol	73	24.59%
*Viral*	*28*	
*NASH*	*19*	
*Medication*	*1*	
*Hemochromatosis*	*10*	
*Combination*	*6*	
*Autoimmune*	*3*	
*Unknown*	*6*	
Yes	225	75.5%
*Cause of cirrhosis: alcohol*	*160*	
*Alcohol and cause of cirrhosis different from alcohol*	*65*	
*Viral*	*5*	
*NASH*	*9*	
*Medication*	*-*	
*Hemochromatosis*	*1*	
*Combination*	*31*	
*Autoimmune*	*-*	
*Unknown*	*19*	
**Pugh-Child** (MD = 82)		
A5	139	58.9%
A6	65	27.5%
B7	19	8.1%
B8	9	3.8%
B9	3	1.3%
C	1	0.4%
**Ascites** (MD = 20)	12	4.0%
**MELD score** (MD = 152)	9	(6; 19)
**Okuda classification** (MD = 105)		
Stage 1	179	84.0%
Stage 2	34	16.0%
**BCLC classification** (MD = 48)		
0	33	12.2%
A	128	47.4%
B	32	11.9%
C	55	20.4%
D	22	8.1%
**Bilirubin in µmol/L** (MD = 85)	13.9	(3.3; 81.0)
**Albumin in g/L** (MD = 101)	37.0	(21.9; 48.0)
**PT as a %** (MD = 75)	82	(44; 110)
**AFP in ng/mL** (MD = 67)	9.8	(0.9; 44,286.0)
**Portal vein thrombosis** (MD = 82)	40	16.9%
**Number of target lesions** (MD = 1)		
1	232	73.2%
2	66	20.8%
3	17	5.4%
4	2	0.6%
**Tumor size in mm**		
Lesion 1 (N = 303)	35	(7; 105)
Lesion 2 (N = 78)	20	(5; 65)
Lesion 3 (N = 17)	12	(5; 23)
Lesion 4 (N = 1)	8	(8; 8)
Total of all lesions (N = 399, MD = 24)	30	(5; 105)
**Previous treatments**		
**Patients who received** **at least one previous treatment** (MD = 3)	111	35.2%
**Previous treatment received**		
Previous local treatment (MD = 3)	100	31.7%
Previous surgery (MD = 2)	16	5.1%
Previous radiofrequency ablation (MD = 2)	28	8.9%
Previous chemoembolization (MD = 3)	68	21.6%
Previous radiotherapy (MD = 2)	2	0.6%
Previous chemotherapy (MD = 2)	2	0.6%
Previous targeted therapy (MD = 2)	23	7.3%

Frequencies and percent are presented for qualitative data and median and range are presented for quantitative data. Either alcohol or cause of cirrhosis must be at least informed. MD: missing data; ECOG performance status: performance status according to the Eastern Cooperative Oncology Group score; BMI: body mass index; NASH: non-alcoholic steatohepatitis; MELD: model for end-stage liver disease; BCLC: Barcelona Clinic Liver Cancer; PT: prothrombin time; AFP: alpha foetoprotein. For the previous treatment received, several types of treatment could be combined for the same patient.

**Table 2:** Description of Cyberknife treatment and dosimetry data.

Cyberknife Treatment Characteristics		
**RT duration in days**	7	(0; 28)
**The total dose in Gy** (MD = 2)	45	(21; 54)
**Number of fractions** (MD = 2)		
3	299	94.6%
4	1	0.3%
5	2	0.6%
6	14	4.4%
**Dose per fraction in Gy** (MD = 2)	15.0	(3.5; 15.0)
**Total dose received in Gy** (MD = 2)	45	(15; 54)
**Early treatment discontinuation** (MD = 2)	2	0.6%
**Liver volume** (MD = 110)	1581.5	(743.6; 3316.7)
**Dmax 700 cc liver** (MD = 111)	9.2	(0.4; 34.1)
**PTV/total liver volume ratio (%)** (MD = 21)	7.2	(0.5; 44.4)
<10%	196	66.0%
≥10%	101	34.0%
**Dosimetric data (N = 375 lesions)**		
**GTV in cm^3^** (MD = 1)	21.1	(0.4; 700.1)
**CTV in cm^3^** (MD = 15)	63.3	(2.5; 999.4)
**PTV in cm^3^** (MD = 9)	90.7	(2.6; 1067.6)
**D2% of GTV in Gy** (MD = 8)	52.4	(25.2; 76.3)
**D50% of GTV in Gy** (MD = 8)	50.0	(23.9; 68.4)
**D98% of GTV in Gy** (MD = 8)	46.1	(21.1; 61.9)
**D2% of CTV in Gy** (MD = 22)	52.5	(27.6; 75.2)
**D50% of CTV in Gy** (MD = 22)	49.7	(25.5; 66.1)
**D98% of CTV in Gy** (MD = 22)	45.6	(14.9; 58.3)
**D2% of PTV in Gy** (MD = 16)	52.3	(25.1; 74.8)
**D50% of PTV in Gy** (MD = 16)	48.9	(23.4; 63.2)
**D98% of PTV in Gy** (MD = 16)	43.6	(13.3; 52.4)

Frequencies and percent are presented for qualitative data and median and range are presented for quantitative data. RT: radiotherapy; Gy: gray; MD: missing data; PTV: planning target volume; GTV: gross tumor volume; CTV: clinical target volume.

**Table 3:** Description of observed acute toxicities with grades in columns (N = 318).

Type of Acute Toxicity	Toxicity (N, %)	Grade ≥ 2 Toxicity	G1	G2	G3
**Toxicity of all types combined (MD = 2)**	**161 (50.9%)**	**53 (16.8%)**	**108**	**45**	**8**
***Fatigue (MD = 9)***	79 (25.6%)	22 (7.1%)	57	22	-
***Pain (MD = 5)***	31 (9.9%)	5 (1.6%)	26	4	1
***Nausea (MD = 10)***	47 (15.3%)	8 (2.6%)	39	8	-
***Vomiting (MD = 5)***	22 (7.0%)	-	22	-	-
***Hepatitis (MD = 40)***	15 (5.4%)	2 (0.7%)	13	2	-
***Digestive ulceration (MD = 7)***	2 (0.6%)	2 (0.6%)	-	2	-
***Ascitis (MD = 5)***	28 (9.1%)	21 (6.7%)	7	14	7

**Table 4:** Association between overall survival, acute toxicity, and patient/treatment characteristics.

Univariate Analyses	Overall Survival (N = 318)				Acute Toxicity Grade ≥ 2 (N = 316)			
Characteristics	n/N	HR	95% CI	*p*-Value	n/N	OR	95% CI	*p*-Value
**Cause of cirrhosis** (MD = 45)				0.76				0.49
Alcohol	120/160	1			25/160	1		
Viral	28/33	1.09	(0.72–1.65)		8/33	1.73	(0.70–4.26)	
NASH	17/28	0.86	(0.52–1.43)		3/28	0.64	(0.18–2.31)	
Other	40/52	1.12	(0.79–1.63)		10/51	1.32	(0.58–2.97)	
**Cirrhosis-alcohol** (MD = 20)				0.13				0.25
No	57/73	1			15/73	1		
Yes	168/225	0.79	(0.59–1.07)		33/223	0.67	(0.34–1.32)	
**Pugh-Child** (MD = 82)				0.003				0.31
A	143/204	1			31/203	1		
B-C	28/32	1.87	(1.25–2.82)		7/31	1.61	(0.64–4.08)	
**MELD score** (MD = 152)				0.27				0.13
/1 unit	-	1.04	(0.97–1.11)		-	1.12	(0.97–1.30)	
**Pretreatment bilirubin** (MD = 85)				0.09				0.02
/10 µmol/L	-	1.12	(0.98–1.27)		-	1.31	(1.04–1.65)	
**Pretreatment albumin** (MD = 101)				0.07				0.61
/10 g/L	-	0.74	(0.54–1.03)		-	0.82	(0.39–1.73)	
**Pretreatment AFP** (MD = 67)				0.103				0.83
/10^3^ ng/mL	-	1.02	(0.99–1.05)		-	0.99	(0.89–1.10)	
**Portal vein thrombosis** (MD = 82)				0.02				0.89
No	133/196	1			31/195	1		
Yes	39/40	1.56	(1.09–2.23)		6/40	0.93	(0.36–2.41)	
**BCLC classification** (MD = 48)				<0.001				0.88
0	20/33	1			4/33	1		
A	87/128	1.31	(0.80–2.13)		22/128	1.50	(0.48–4.71)	
B	25/32	1.52	(0.84–2.74)		6/32	1.67	(0.42–6.59)	
C	45/55	1.89	(1.11–3.21)		7/54	1.08	(0.29–4.01)	
D	18/22	3.64	(1.92–6.93)		4/21	1.71	(0.38–7.72)	
**Total GTV in cm^3^** (MD = 20)				<0.001				0.02
/100 cm3	-	1.22	(1.09–1.36)		-	1.34	(1.04–1.73)	
**Total GTV in cm^3^** (MD = 20)				<0.001				
Quartile 1–3 (<70)	159/224	1						
Quartile 4 (>70)	64/74	1.81	(1.34–2.42)					
**PTV/total liver vol ratio** (%) (MD = 21)				<0.001				0.004
/10%	-	1.34	(1.17–1.55)		-	1.63	(1.17–2.26)	

Acute toxicity data were missing for two patients. Total GTV in cm^3^ was considered in two categories according to the third quartile for overval survival because of similar HR for the first three quartiles (quartile 1, the reference; quartile 2: HR = 1.14, 95%CI 0.78–1.67; quartile 3: HR = 0.99, 95%CI 0.67–1.46). PTV/total volume liver ratio was considered as continuous for acute toxicity because of OR increasing monotonously across the quartiles (quartile 1, the reference; quartile 2: HR = 1.54, quartile 3: HR = 1.38, quartile 4: HR = 1.82). HR: hazard ratio; OR: odds ratio; MD: missing data; NASH: non-alcoholic steatohepatitis; MELD: model for end-stage liver disease; AFP: alpha fetoprotein; BCLC: Barcelona Clinic Liver Cancer; GTV: gross tumor volume; PTV: planning target volume.

## Data Availability

The study complies with the “reference methodology” adopted by the French Data Protection Authority (CNIL).

## References

[1] Cancer Today. http://gco.iarc.fr/today/home.

[2] European Association for the Study of the Liver (2018). EASL Clinical Practice Guidelines: Management of hepatocellular carcinoma. J. Hepatol..

[3] Heimbach J.K., Kulik L.M., Finn R.S., Sirlin C.B., Abecassis M.M., Roberts L.R., Zhu A.X., Murad M.H., Marrero J.A. (2018). AASLD guidelines for the treatment of hepatocellular carcinoma. Hepatology.

[4] Llovet J.M., Bruix J. (2003). Systematic review of randomized trials for unresectable hepatocellular carcinoma: Chemoembolization improves survival. Hepatology.

[5] Bruix J., Sherman M. (2005). Management of hepatocellular carcinoma. Hepatology.

[6] Bruix J., Sherman M. (2011). Management of hepatocellular carcinoma: An update. Hepatology.

[7] Llovet J.M., Ricci S., Mazzaferro V., Hilgard P., Gane E., Blanc J.-F., De Oliveira A.C., Santoro A., Raoul J.-L., Forner A. (2008). Sorafenib in Advanced Hepatocellular Carcinoma. N. Engl. J. Med..

[8] Kudo M., Finn R.S., Qin S., Han K.-H., Ikeda K., Piscaglia F., Baron A., Park J.-W., Han G., Jassem J. (2018). Lenvatinib versus sorafenib in first-line treatment of patients with unresectable hepatocellular carcinoma: A randomised phase 3 non-inferiority trial. Lancet.

[9] Boige V., Barbare J.-C., Rosmorduc O. (2008). Utilisation du sorafénib (Nexavar^®^) dans le traitement du carcinome hépatocellulaire: Recommandations Prodige Afef. Gastroentérologie Clin. Biol..

[10] El-Khoueiry A.B., Sangro B., Yau T., Crocenzi T.S., Kudo M., Hsu C., Kim T.-Y., Choo S.-P., Trojan J., Welling T.H. (2017). Nivolumab in patients with advanced hepatocellular carcinoma (CheckMate 040): An open-label, non-comparative, phase 1/2 dose escalation and expansion trial. Lancet.

[11] Zhu A.X., Finn R.S., Edeline J., Cattan S., Ogasawara S., Palmer D., Verslype C., Zagonel V., Fartoux L., Vogel A. (2018). Pembrolizumab in patients with advanced hepatocellular carcinoma previously treated with sorafenib (KEYNOTE-224): A non-randomised, open-label phase 2 trial. Lancet Oncol..

[12] Finn R.S., Qin S., Ikeda M., Galle P.R., Ducreux M., Kim T.-Y., Kudo M., Breder V., Merle P., Kaseb A.O. (2020). Atezolizumab plus Bevacizumab in Unresectable Hepatocellular Carcinoma. N. Engl. J. Med..

[13] Durand-Labrunie J., Baumann A.-S., Ayav A., Laurent V., Boleslawski E., Cattan S., Bogart E., Le Deley M.-C., Steen V., Lacornerie T. (2020). Curative Irradiation Treatment of Hepatocellular Carcinoma: A Multicenter Phase 2 Trial. Int. J. Radiat. Oncol. Biol. Phys..

[14] Andolino D.L., Johnson C.S., Maluccio M., Kwo P., Tector A.J., Zook J., Johnstone P.A., Cardenes H.R. (2011). Stereotactic Body Radiotherapy for Primary Hepatocellular Carcinoma. Int. J. Radiat. Oncol. Biol. Phys..

[15] Lo C.-H., Yang J.-F., Liu M.-Y., Jen Y.-M., Lin C.-S., Chao H.-L., Huang W.-Y. (2017). Survival and prognostic factors for patients with advanced hepatocellular carcinoma after stereotactic ablative radiotherapy. PLoS ONE.

[16] Que J., Kuo H.-T., Lin L.-C., Lin K.-L., Lin C.-H., Lin Y.-W., Yang C.-C. (2016). Clinical outcomes and prognostic factors of cyberknife stereotactic body radiation therapy for unresectable hepatocellular carcinoma. BMC Cancer.

[17] Park S., Jung J., Cho B., Kim S.Y., Yun S., Lim Y., Lee H.C., Park J., Park J., Kim J.H. (2020). Clinical outcomes of stereotactic body radiation therapy for small hepatocellular carcinoma. J. Gastroenterol. Hepatol..

[18] Llovet J.M., Fuster J., Bruix J., Barcelona-Clínic Liver Cancer Group (2004). The Barcelona approach: Diagnosis, staging, and treatment of hepatocellular carcinoma. Liver Transpl..

[19] Jarraya H., Chalayer C., Tresch E., Bonodeau F., Lacornerie T., Mirabel X., Boulanger T., Taieb S., Kramar A., Lartigau E. (2014). Novel Technique for Hepatic Fiducial Marker Placement for Stereotactic Body Radiation Therapy. Int. J. Radiat. Oncol. Biol. Phys..

[20] Benedict S.H., Yenice K.M., Followill D., Galvin J.M., Hinson W., Kavanagh B., Keall P., Lovelock M., Meeks S., Papiez L. (2010). Stereotactic body radiation therapy: The report of AAPM Task Group 101. Med. Phys..

[21] ICRU Report 91. Prescribing, Recording, and Reporting of Stereotactic Treatments with Small Photon Beams—ICRU. https://www.icru.org/report/icru-report-91-prescribing-recording-and-reporting-of-stereotactic-treatments-with-small-photon-beams-2/.

[22] Lencioni R., Llovet J.M. (2010). Modified RECIST (mRECIST) Assessment for Hepatocellular Carcinoma. Semin. Liver Dis..

[23] Kokudo N., Takemura N., Hasegawa K., Takayama T., Kubo S., Shimada M., Nagano H., Hatano E., Izumi N., Kaneko S. (2019). Clinical practice guidelines for hepatocellular carcinoma: The Japan Society of Hepatology 2017 (4th JSH-HCC guidelines) 2019 update. Hepatol. Res..

[24] Omata M., Cheng A.-L., Kokudo N., Kudo M., Lee J.M., Jia J., Tateishi R., Han K.-H., Chawla Y.K., Shiina S. (2017). Asia–Pacific clinical practice guidelines on the management of hepatocellular carcinoma: A 2017 update. Hepatol. Int..

[25] Vogel A., Cervantes A., Chau I., Daniele B., Llovet J.M., Meyer T., Nault J.-C., Neumann U., Ricke J., Sangro B. (2018). Hepatocellular carcinoma: ESMO Clinical Practice Guidelines for diagnosis, treatment and follow-up. Ann. Oncol..

[26] Dobrzycka M., Spychalski P., Rostkowska O., Wilczyński M., Kobiela P., Grąt M., Dell’Acqua V., Høyer M., Jereczek-Fossa B.A. (2019). Stereotactic body radiation therapy for early-stage hepatocellular carcinoma—A systematic review on outcome. Acta Oncol..

[27] Ohkoshi-Yamada M., Kamimura K., Shibata O., Morita S., Kaidu M., Nakano T., Maruyama K., Ota A., Saito H., Yamana N. (2020). Efficacy and Safety of the Radiotherapy for Liver Cancer: Assessment of Local Controllability and its Role in Multidisciplinary Therapy. Cancers.

[28] Bibault J.-E., Dewas S., Vautravers-Dewas C., Hollebecque A., Jarraya H., Lacornerie T., Lartigau E., Mirabel X. (2013). Stereotactic Body Radiation Therapy for Hepatocellular Carcinoma: Prognostic Factors of Local Control, Overall Survival, and Toxicity. PLoS ONE.

[29] Jang W.I., Bae S.H., Kim M., Han C.J., Park S.C., Kim S.B., Cho E., Choi C.W., Kim K.S., Hwang S. (2019). A phase 2 multicenter study of stereotactic body radiotherapy for hepatocellular carcinoma: Safety and efficacy. Cancer.

[30] Bujold A., Massey C.A., Kim J.J., Brierley J., Cho C., Wong R.K., Dinniwell R.E., Kassam Z., Ringash J., Cummings B. (2013). Sequential Phase I and II Trials of Stereotactic Body Radiotherapy for Locally Advanced Hepatocellular Carcinoma. J. Clin. Oncol..

[31] Takeda A., Sanuki N., Tsurugai Y., Iwabuchi S., Matsunaga K., Ebinuma H., Imajo K., Aoki Y., Saito H., Kunieda E. (2016). Phase 2 study of stereotactic body radiotherapy and optional transarterial chemoembolization for solitary hepatocellular carcinoma not amenable to resection and radiofrequency ablation. Cancer.

[32] Lee J., Shin I.-S., Yoon W.S., Koom W.S., Rim C.H. (2020). Comparisons between radiofrequency ablation and stereotactic body radiotherapy for liver malignancies: Meta-analyses and a systematic review. Radiother. Oncol..

[33] Pan Y.-X., Fu Y.-Z., Hu D.-D., Long Q., Wang J.-C., Xi M., Liu S.-L., Xu L., Liu M.-Z., Chen M.-S. (2020). Stereotactic Body Radiotherapy vs. Radiofrequency Ablation in the Treatment of Hepatocellular Carcinoma: A Meta-Analysis. Front. Oncol..

[34] Wang L., Ke Q., Huang Q., Shao L., Chen J., Wu J. (2020). Stereotactic body radiotherapy versus radiofrequency ablation for hepatocellular carcinoma: A systematic review and meta-analysis. Int. J. Hyperth..

[35] Bettinger D., Gkika E., Schultheiss M., Glaser N., Lange S., Maruschke L., Buettner N., Kirste S., Nestle U., Grosu A.-L. (2018). Comparison of local tumor control in patients with HCC treated with SBRT or TACE: A propensity score analysis. BMC Cancer.

[36] Su T.-S., Liang P., Zhou Y., Huang Y., Cheng T., Qu S., Chen L., Xiang B.-D., Zhao C., Huang D.-J. (2020). Stereotactic Body Radiation Therapy vs. Transarterial Chemoembolization in Inoperable Barcelona Clinic Liver Cancer Stage a Hepatocellular Carcinoma: A Retrospective, Propensity-Matched Analysis. Front. Oncol..

[37] Sapir E., Tao Y., Schipper M.J., Bazzi L., Novelli P.M., Devlin P., Owen D., Cuneo K.C., Lawrence T.S., Parikh N.D. (2018). Stereotactic Body Radiation Therapy as an Alternative to Transarterial Chemoembolization for Hepatocellular Carcinoma. Int. J. Radiat. Oncol. Biol. Phys..

[38] Shen P.-C., Chang W.-C., Lo C.-H., Yang J.-F., Lee M.-S., Dai Y.-H., Lin C.-S., Fan C.-Y., Huang W.-Y. (2019). Comparison of Stereotactic Body Radiation Therapy and Transarterial Chemoembolization for Unresectable Medium-Sized Hepatocellular Carcinoma. Int. J. Radiat. Oncol. Biol. Phys..

[39] Su T.-S., Liang P., Liang J., Lu H.-Z., Jiang H.-Y., Cheng T., Huang Y., Tang Y., Deng X. (2017). Long-Term Survival Analysis of Stereotactic Ablative Radiotherapy Versus Liver Resection for Small Hepatocellular Carcinoma. Int. J. Radiat. Oncol. Biol. Phys..

[40] Kim M., Kay C.S., Jang W.I., Kim M.-S., Lee D.S., Jang H.S. (2017). Prognostic value of tumor volume and radiation dose in moderate-sized hepatocellular carcinoma. Medicine.

[41] Mathew A.S., Atenafu E.G., Owen D., Maurino C., Brade A., Brierley J., Dinniwell R., Kim J., Cho C., Ringash J. (2020). Long term outcomes of stereotactic body radiation therapy for hepatocellular carcinoma without macrovascular invasion. Eur. J. Cancer.

[42] Zhang L., Yan L., Niu H., Ma J., Yuan B.-Y., Chen Y.-H., Zhuang Y., Hu Y., Zeng Z.-C., Xiang Z.-L. (2019). A nomogram to predict prognosis of patients with unresected hepatocellular carcinoma undergoing radiotherapy: A population-based study. J. Cancer.

[43] Liu H.Y., Lee Y., McLean K., Leggett D., Hodgkinson P., Fawcett J., Mott R., Stuart K., Pryor D. (2020). Efficacy and Toxicity of Stereotactic Body Radiotherapy for Early to Advanced Stage Hepa-tocellular Carcinoma—Initial Experience from an Australian Liver Cancer Service. Clin. Oncol..

[44] Finn R.S., Ryoo B.-Y., Merle P., Kudo M., Bouattour M., Lim H.Y., Breder V., Edeline J., Chao Y., Ogasawara S. (2020). Pembrolizumab as Second-Line Therapy in Patients with Advanced Hepatocellular Carcinoma in KEYNOTE-240: A Randomized, Double-Blind, Phase III Trial. J. Clin. Oncol..

[45] Jagodinsky J.C., Harari P.M., Morris Z.S. (2020). The Promise of Combining Radiation Therapy with Immunotherapy. Int. J. Radiat. Oncol. Biol. Phys..

[46] Kreidieh M., Zeidan Y.H., Shamseddine A. (2019). The Combination of Stereotactic Body Radiation Therapy and Immunotherapy in Primary Liver Tumors. J. Oncol..

[47] Lee Y.H., Tai D., Yip C., Choo S.P., Chew V. (2020). Combinational Immunotherapy for Hepatocellular Carcinoma: Radiotherapy, Immune Checkpoint Blockade and Beyond. Front. Immunol..

[48] Chiang C.-L., Chan A.C.Y., Chiu K.W.H., Kong F.-M. (2019). Combined Stereotactic Body Radiotherapy and Checkpoint Inhibition in Unresectable Hepatocellular Carcinoma: A Potential Synergistic Treatment Strategy. Front. Oncol..

[49] Pérez-Romasanta L.A., Portillo E.G.-D., Rodríguez-Gutiérrez A., Matías-Pérez Á. (2021). Stereotactic Radiotherapy for Hepatocellular Carcinoma, Radiosensitization Strategies and Radiation-Immunotherapy Combination. Cancers.

[50] Mazzaferro V., Bhoori S., Sposito C., Bongini M., Langer M., Miceli R., Mariani L. (2011). Milan criteria in liver transplantation for hepatocellular carcinoma: An evidence-based analysis of 15 years of experience. Liver Transplant..

[51] Wang Y.-F., Dai Y.-H., Lin C.-S., Chang H.-C., Shen P.-C., Yang J.-F., Hsiang C.-W., Lo C.-H., Huang W.-Y. (2021). Clinical outcome and pathologic correlation of stereotactic body radiation therapy as a bridge to transplantation for advanced hepatocellular carcinoma: A case series. Radiat. Oncol..

[52] Mannina E.M., Cardenes H.R., Lasley F.D., Goodman B., Zook J., Althouse S., Cox J.A., Saxena R., Tector J., Maluccio M. (2017). Role of Stereotactic Body Radiation Therapy before Orthotopic Liver Trans-plantation: Retrospective Evaluation of Pathologic Response and Outcomes. Int. J. Radiat. Oncol. Biol. Phys..

[53] Wong T.C., Lee V.H., Law A.L., Pang H.H., Lam K., Lau V., Cui T.Y., Fong A.S., Lee S.W., Wong E.C. (2021). Prospective Study of Stereotactic Body Radiation Therapy for Hepatocellular Carcinoma on Waitlist for Liver Transplant. Hepatology.

[54] Lee P., Ma Y., Zacharias I., Bozorgzadeh A., Wilson S., Foley K., Rava P., Masciocchi M., Ding L., Bledsoe J. (2020). Stereotactic Body Radiation Therapy for Hepatocellular Carcinoma in Patients with Child-Pugh B or C Cirrhosis. Adv. Radiat. Oncol..

[55] Gresswell S., Tobillo R., Hasan S., Uemura T., Machado L., Thai N., Kirichenko A. (2018). Stereotactic body radiotherapy used as a bridge to liver transplant in patients with hepatocellular carcinoma and Child-Pugh score ≥ 8 cirrhosis. J. Radiosurg. SBRT.

